# Effective inter-residue contact definitions for accurate protein fold recognition

**DOI:** 10.1186/1471-2105-13-292

**Published:** 2012-11-09

**Authors:** Chao Yuan, Hao Chen, Daisuke Kihara

**Affiliations:** 1 Department of Biological Sciences, Purdue University, West Lafayette, IN, 47907, USA; 2 Department of Computer Science, Purdue University, West Lafayette, IN, 47907, USA

**Keywords:** Protein structure prediction, Threading, Fold recognition, Structural features, Residue-residue contact, Protein fold

## Abstract

**Background:**

Effective encoding of residue contact information is crucial for protein structure prediction since it has a unique role to capture long-range residue interactions compared to other commonly used scoring terms. The residue contact information can be incorporated in structure prediction in several different ways: It can be incorporated as statistical potentials or it can be also used as constraints in ab initio structure prediction. To seek the most effective definition of residue contacts for template-based protein structure prediction, we evaluated 45 different contact definitions, varying bases of contacts and distance cutoffs, in terms of their ability to identify proteins of the same fold.

**Results:**

We found that overall the residue contact pattern can distinguish protein folds best when contacts are defined for residue pairs whose Cβ atoms are at 7.0 Å or closer to each other. Lower fold recognition accuracy was observed when inaccurate threading alignments were used to identify common residue contacts between protein pairs. In the case of threading, alignment accuracy strongly influences the fraction of common contacts identified among proteins of the same fold, which eventually affects the fold recognition accuracy. The largest deterioration of the fold recognition was observed for β-class proteins when the threading methods were used because the average alignment accuracy was worst for this fold class. When results of fold recognition were examined for individual proteins, we found that the effective contact definition depends on the fold of the proteins. A larger distance cutoff is often advantageous for capturing spatial arrangement of the secondary structures which are not physically in contact. For capturing contacts between neighboring β strands, considering the distance between Cα atoms is better than the Cβ−based distance because the side-chain of interacting residues on β strands sometimes point to opposite directions.

**Conclusion:**

Residue contacts defined by Cβ−Cβ distance of 7.0 Å work best overall among tested to identify proteins of the same fold. We also found that effective contact definitions differ from fold to fold, suggesting that using different residue contact definition specific for each template will lead to improvement of the performance of threading.

## Background

The tertiary structure of proteins provides crucial information for understanding molecular mechanisms of biological functions. Protein structures also serve as a platform for various branches of biotechnology, including drug design [1,2] and protein engineering [3-5]. Although protein structures have been solved by experiments at an increasing rate, a flood of new sequences have been determined even more rapidly due to the advance of sequencing technologies [6,7]. Taking advantage of the enlarging database of experimentally solved protein structures [8], it is expected that computational structure prediction methods, especially template-based methods, will play a more significant role in providing structure of newly sequenced proteins [9-12]. However, computing accurate structure models is still not always possible especially when template structures available do not share significant sequence similarity to a target sequence [13]. Template-based structure prediction methods usually employ structure-based scoring terms together with sequence matching terms to enhance structure recognition and alignment accuracy [14-18]. Structure-based terms used include secondary structure prediction [19], main-chain angle propensity [20], burial/exposure status [19], residue depth [15], and the number of residue contacts [16] for each amino acid. These structure-based terms are commonly derived from statistics of structural properties observed in representative structures (knowledge-based statistical potentials). Among various structure-based terms, residue-residue contact potentials [21-23] are unique in that they capture long-range interactions in a protein structure [24]. A proper encoding of residue contact information is crucial for structure prediction because in principle, a full distance map or a residue contact map has sufficient information for reconstructing the tertiary structure of a protein [25]. It has been also shown that a certain fraction of errors or missing contacts are tolerated for modeling the native structure of proteins [26-28]. When contact information is used as constraints in an “*ab initio*” structure prediction method, even very sparse information of residue contacts, for example, a contact for every eight residues in a protein sequence is sufficient to reconstruct the native structure [29]. Correct identification of residue contacts is also important for template-based structure prediction since contact maps are usually well conserved between proteins of the same fold even at a very low sequence identity [30]. There are two strategies of using residue contact information for structure prediction. One is to predict residue contact from a protein sequence [31-37] and use them as constraints or as an additional scoring term in a structure prediction procedure [38]. The other approach is to employ a knowledge-based statistical residue contact potential to take into account general propensity of residue interactions. Various types of contact potentials have been proposed and applied for protein structure prediction [21-23,39,40]. They share the same principle but vary in details of their designs. For example, they differ in the definition of residue contacts, the reference state, whether or not to consider dependency to the distance and orientation. There are also contact potentials that consider more than two residues that are in contact [41,42]. Here, we examined various definitions of residue contacts to identify the most effective definitions in the context of fold recognition. In contrast to the previous works that evaluated contact maps in terms of the accuracy of protein structure reconstruction [26-28], we examine definitions of residue contacts that can effectively distinguish proteins of the same fold from those of the other folds. Thus, information contained in residue contacts that are specific to each protein fold is evaluated in purely a practical scenario of the fold recognition.

Concretely, we prepared 45 different contact definitions that consist of combinations of three different contacting atoms, i.e. Cα, Cβ, and heavy atoms with 15 distance cutoffs. Using the 45 different contact definitions, we examined how well contact maps defined by each definition can distinguish proteins of the same fold from others. The similarity of contact maps of two proteins is defined as the fraction of the common contacts between the two proteins, where equivalent residues are identified either by structural superimposition or a threading method. The purpose of using threading methods is to simulate the actual situation of threading where an alignment between a query sequence and a template structure is not always accurate. We found that 7.5/7.0 Å, 7.0/6.5 Å, and 4.5/5.0 Å perform best for the distance cutoff of contact definition using Cα, Cβ, and heavy atoms, respectively, for identifying protein pairs of the same fold. These cutoffs worked consistently well when threading-based alignments were used for identifying equivalent residues in protein pairs. On average, contact maps effectively distinguish proteins of the same fold from others when contacting residue pairs occupy 4.1 – 6.9% of the whole contact maps. We also found that effective contact definitions differ from fold to fold, suggesting that using different residue contact definition specific for each template will lead to improvement of threading performance.

## Results

### Structural retrieval performance using different contact definitions

**Figure 1:**
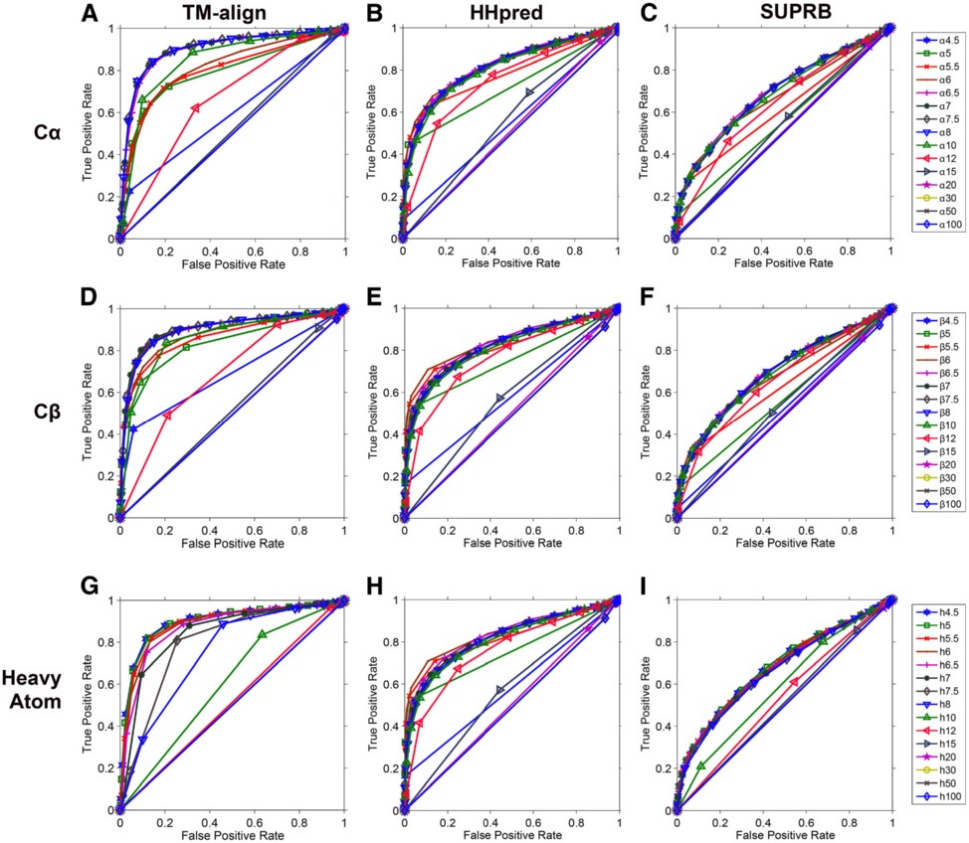
**ROC curves of structure pair retrieval on the fold level dataset.** To determine corresponding residues in protein pairs, TM-align (the left column), HHpred (the middle column), and SUPRB (the right column) were used. Three different bases for residue contact definitions are used, the Cα-Cα distance (the first row), the Cβ-Cβ distance (the second row), and the heavy atom distance (the third row). **A**, ROC curves using TM-align alignments and the Cα-Cα distance for contact definition. 15 different distance cutoffs were used, namely, 4.5, 5.0, 5.5, 6.0, 6.5, 7.0, 7.5, 8.0, 10.0, 12.0, 15.0, 20.0, 30.0, 50.0, and 100.0 Å to define contacts. **B**, HHpred alignments and the Cα-Cα distance were used. **C**, SUPRB alignments and the Cα-Cα distance were used. **D**, ROC curves using TM-align alignments and the Cβ-Cβ distance for contact definition. The same 15 different distance thresholds as used for the Cα-Cα distance cutoffs were used. **E**, HHpred alignments and the Cβ-Cβ distance were used. **F**, SUPRB alignments and the Cβ-Cβ distance were used. **G**, ROC curves using TM-align alignments and the heavy atom distance for contact definition. The same 15 different distance thresholds as used for the Cα-Cα distance cutoffs were used. **H**, HHpred alignments and the heavy atom distance were used. **I**, SUPRB alignments and the heavy atom distance were used

In Figure 1 we show structural retrieval performance by considering the fraction of common contacts (FCC) of protein pairs, which is computed using 45 different residue contact definitions. For a protein fold, protein pairs within the same fold and across different folds were ranked according to their FCC values, with which ROC curves were plotted. To identify equivalent residues between proteins, we used TM-align [43], a structural superimposition program (Figures 1A, 1D, 1G), as well as two threading methods, HHpred [44] (Figures 1B, 1E, 1H) and SUPRB [12] (Figures 1C, 1F, 1I). The 45 different contact definitions consist of 15 distance cutoffs (4.5, 5.0, 5.5, 6.0, 6.5, 7.0, 7.5, 8.0, 10.0, 12.0, 15.0, 20.0, 30.0, 50.0, and 100.0 Å) for distances between Cα-Cα, Cβ–Cβ, and heavy atoms of residue pairs. 

When equivalent residues between structure pairs were correctly identified by structural alignments (TM-align) and thus FCC was accurately computed, distant cutoffs of 7.5 Å, 7.0 Å, and 4.5 Å showed best discrimination between within- and across-fold pairs for the Cα-Cα (Figure 1A), the Cβ-Cβ (Figure 1D), and the heavy atom (Figure 1G) distances, respectively. Area Under Curve (AUC) for them were 0.908, 0.909, and 0.905, respectively. The other similar cutoffs showed slightly worse but comparable AUC: for the Cα-Cα distance, both 6.5 and 7.0 Å obtained AUC of 0.907. For the Cβ-Cβ, 6.5 and 7.5 Å showed 0.907 and 0.904 AUC, respectively. For the heavy atom distance, 5.0 Å showed 0.903 AUC. Proteins in the same fold become less distinguishable when smaller or larger distance cutoffs were used for residue contact patterns. For the Cα-Cα or the Cβ-Cβ distances, AUC of ROC curves quickly decreased when the 12 Å or larger cutoff was used, reaching to the random retrieval at 20 Å or higher. Using a smaller cutoff, 4.5 Å, also deteriorated the retrieval since contact maps became too sparse (on average only 1.61% or 0.33% of residue pairs in a protein were defined as in contact for the Cα-Cα and the Cβ-Cβ distances, respectively). Using the heavy atom distance, the 4.5 Å cutoff had the highest AUC value. The average occupancy of contact maps with the heavy atom distance of 4.5 Å is 6.71%. This value is comparable to those of the best contact cutoffs for the Cα-Cα distance 8 Å and for the Cβ-Cβ distance 6.5 Å, which are 6.89% and 4.10%, respectively. Later we will investigate the relationship of the occupancy of contact maps and the AUC values more thoroughly. The AUC values by best performing definitions are summarized in Table 1. In terms of the AUC, Cβ 7.0 Å achieved the highest value (0.909), although best performing cutoffs for Cα and Cβ showed similar values (~0.91) (Table 1).

**Table 1:** AUC values of the best contact cutoff values for the three alignment methods

	**TM-align**	**HHpred**	**SUPRB**
Cα 6.5/7.0Å	0.907/0.907	0.843/0.835	0.718/0.712
Cβ 6.5/7.0Å	0.907/0.909	0.847/0.847	0.717/0.720
Heavy atom 4.5/5.0 Å	0.905/0.903	0.836/0.838	0.713/0.713

The AUC value of the ROC curves of the best contact distance cutoffs for the Cα-Cα, the Cβ-Cβ, and the heavy atom distance.

### Structure retrieval with common contacts when threading alignments were used

**Figure 2:**
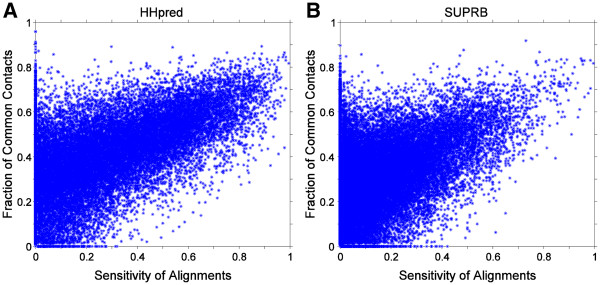
**Correlation between alignment accuracy and the fraction of common contacts (FCC).** The Cβ-Cβ distance with cutoff 6.5Å was used to define residue contact since it demonstrated one of the best fold recognition accuracies. For each pair of proteins of the same fold, the FCC is plotted relative to the alignment sensitivity, which is defined as the fraction of the correctly aligned residue pairs by **A**, HHpred; **B**, SUPRB; among the residue pairs aligned in the correct alignment by TM-Align

**Figure 3:**
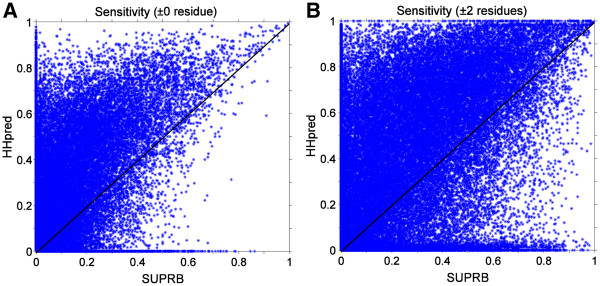
**Comparison of the alignment sensitivity by HHpred and SUPRB. Protein pairs of the same fold were used. A**, an aligned residue pair by HHpred/SUPRB is counted as correct if the pair is also aligned in the alignment by TM-align. **B**, an aligned pair is considered as correct if it is within two residue shift from an aligned pair in the TM-align alignment

The middle and the right column in Figure 1 show structure retrieval results obtained when the threading methods, HHpred (Figures 1B, 1E, 1H) and SUPRB (Figures 1C, 1F, 1I), were used to correspond residues of protein pairs to compare contact maps. The purpose of employing the threading methods is to introduce realistic errors in alignments of protein pairs, which are expected in actual threading process. Overall, lower AUC values were observed when threading methods were used relative to the cases when structural alignments were used (i.e. the left column in Figure 1) for identifying equivalent residues. This deterioration of AUC values is due to the inaccuracy in the threading alignments. As shown in Figure 2, the alignment accuracy (more precisely, the fraction of residues in a query protein that are correctly aligned to a template) and the FCC values correlate with each other for proteins of the same fold. The Pearson’s correlation coefficients for HHpred (Figure 2A) and SUPRB (Figure 2B) are 0.686 and 0.478, respectively. Thus, the more accurate the alignment is for a protein pair in the same fold, the higher FCC is obtained, which will be better distinguished from the background distribution of FCC of proteins of different folds. ROC curves for HHpred (the middle column in Figure 1) showed higher AUCs than those of SUPRB (right column). This is also explained by the higher alignment accuracy by HHpred than SUPRB. As shown in Figure 3, in most of the cases, alignments by HHpred are more accurate than those by SUPRB. The average accuracy of alignments of proteins in the same fold by HHpred and SUPRB is 20.38% (36.60%) and 7.93% (20.27%), respectively. In the parentheses, accuracy was shown when residues aligned within two residues from the correct position are counted as accurate. For 58.12% of the alignments, HHpred results were more accurate than SUPRB. When the accuracy calculation is relaxed to ±2 residues, 70.03% of the alignments by HHpred results were more accurate. Turning our attention back to Figure 1, the best performing distance cutoffs for HHpred (the middle column) and SUPRB (the right column) are consistent with those found for the structural alignments. In all the cases, 6.5/7.0/7.5/8.0 Å, 6.0/6.5/7.0Å, 4.5/5.0Å showed the largest AUC for the Cα-Cα, the Cβ-Cβ, and the heavy atom distances. A qualitative difference of the ROC curves for HHpred/SUPRB is that larger distance cutoffs performed well at a similar level as the best performing distance cutoff compared with the ROC curves for TM-align (structural alignments). For example, the 12 Å cutoff for the Cα-Cα and the Cβ-Cβ distance performed similarly to the 6.5 Å cutoff in the case of HHpred/SUPRB, which was not observed for TM-align. Also, the heavy atom distance 4.5 Å performed clearly better than 6.5 Å in TM-align alignments, but quite similarly in the two threading methods. This is because residue contacts identified under a strict definition tend to be easily missed when incorrect alignments by threading methods are used. On the other hand, contact patterns defined with a larger distance cutoff are more tolerant to residue shifts in threading alignments. The same analysis of structure recognition was performed on the superfamily dataset, which gave consistent results (Additional file 1). Since they gave quite similar results, below we will only discuss the results obtained for the fold level dataset.

### Contact map occupancy

**Figure 4:**
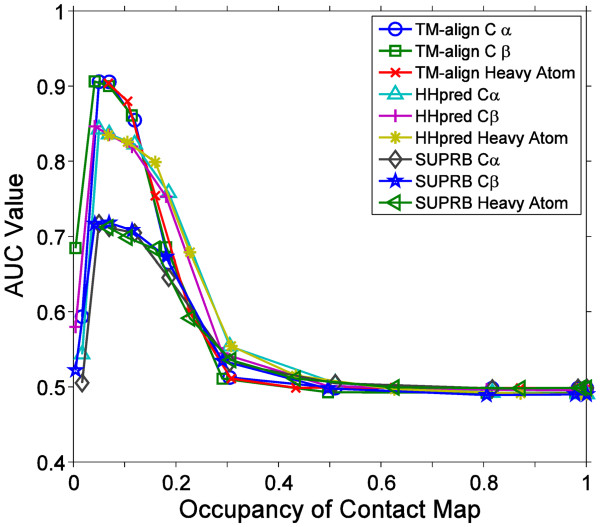
**The average AUC values of structure pair retrieval relative to the average occupancy of contact maps.** Combinations of the three alignment methods and the three bases of residue contacts were examined. The fold level dataset was used. The occupancy of a contact maps is defined by the fraction of the residue pairs in contact under a residue contact definition among all the residue pairs in a protein. For each contact base, the average contact map occupancy and the AUC values are plotted using the ten distance cutoff values

Apparently, contact maps lose fold-specific information if residue contacts are defined with a too short or a too long distance cutoff because maps become too sparse or dense with contacts. To examine how the occupancy of contact map affects the fold retrieval accuracy, we plotted the average AUC values relative to the occupancy of contact maps computed using different distance cutoffs for three different contact bases and the three alignment methods (Figure 4). The occupancy (the x-axis) of a contact map is defined as the fraction of residue pairs in contact among all the pairs of residues in the map. For each combination of a contact base and an alignment method, the highest AUC was observed when the occupancy is at 4.10% to 6.89%. The average AUC slowly drops as the fraction of residue contacts further increases and reaches to random retrieval level when 30-40% of residue pairs are defined as in contact.

### Structural retrieval evaluated with TM-score

**Figure 5:**
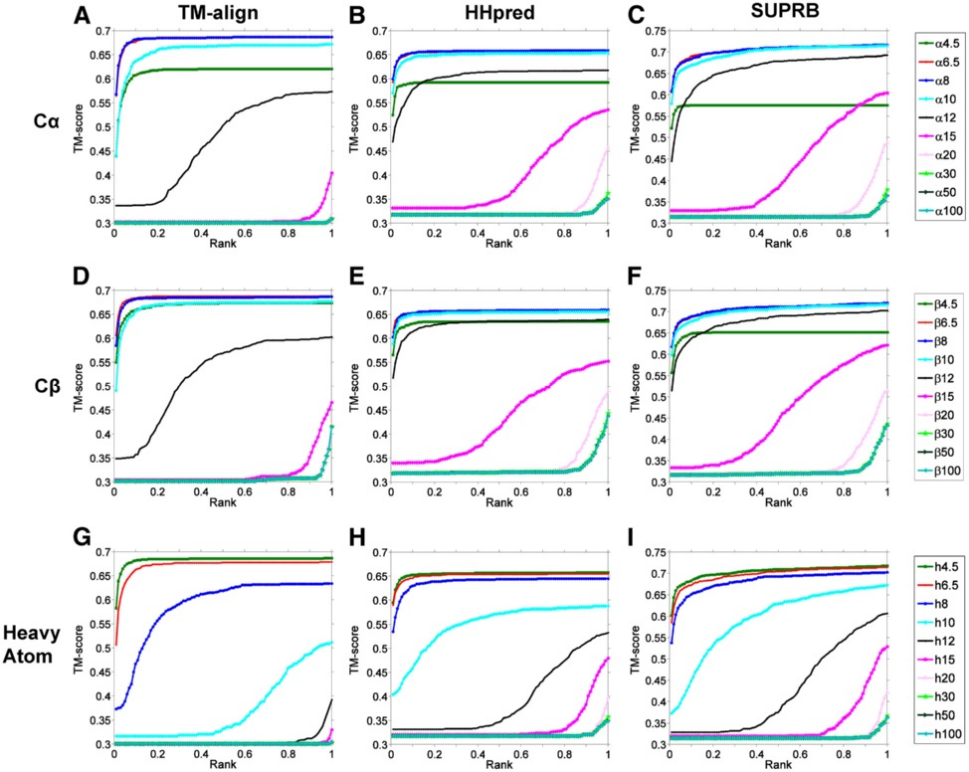
**The best TM-score observed among the top ranked protein pairs.** Protein pairs in the fold level dataset was ranked by their FCC and for each pair (of a query and a template) the structural similarity of the structural models of the query inferred by the alignment was compared with the native structure of the query using TM-align. At each rank in the x-axis, the best TM-score was plotted. For protein pairs with equal FCC, TM-scores are averaged among the pairs. To determine corresponding residues in protein pairs, TM-align (the left column), HHpred (the middle column), and SUPRB (the right column) were used. Three different bases for residue contact definitions are used, the Cα-Cα distance (the first row), the Cβ-Cβ distance (the second row), and the heavy atom distance (the third row). The panels are ordered in the same way as Figure 1

We have also evaluated the retrieval performance in terms of the structural similarity of the top ranked protein pairs. In Figure 5, the best TM-score of protein pairs up to certain ranks are plotted using the contact maps of a subset of residue contact definitions as Figure 1 (4.5, 6.5, 8.0, 10.0 12.0, 15.0, 20.0, 30.0, 50.0, 100.0 Å for the Cα-Cα, the Cβ-Cβ, and the heavy atom distance). The relative performance of each distance cutoff is essentially consistent with the ROC curves in Figure 1. Using 6.5 Å and 8 Å for the Cα-Cα and the Cβ-Cβ distance and 4.5 Å for the heavy atom distance retrieved structurally similar protein pairs at higher ranks than the other distant cutoffs. Using distance cutoffs of 15 Å, 15 Å, 12 Å or larger for the Cα-Cα, the Cβ-Cβ, and the heavy atom distance did not yield protein pairs with significant structural similarity (TM-score > 0.5 [45]) within the earlier half of the ranks in the retrieval. 

### Fold recognition using residue contacts of different sequence separation ranges

**Figure 6:**
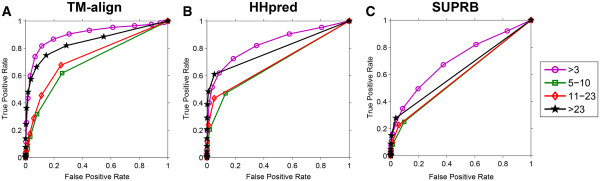
**ROC curves using residue contacts of different sequence separations.** Residue contacts defined by the Cβ-Cβ distance 6.5Å are binned to 5–10, 11–23, and >23 and each of them are used for fold recognition separately. >3 is the result using all the residue contacts (as done for the other figures, short range contacts with 1 to 2 residue separation are not considered). **A**, TM-align, **B**, HHpred, and **C**, SUPRB

In Figure 6, fold recognition was performed using residue contacts with different sequence separation ranges, contacting residue pairs that are separated by 5–10 residues on the sequence, 11–23, and over 23 residues apart. The contact definition of Cβ-Cβ, 6.5 Å was used, since it was one of the best performing definitions for the three alignment methods. Interestingly, the largest AUC value was achieved by using >23 contacts, consistently for TM-Align, HHpred, and SUPRB alignments. AUC values for 5–10, 11–23, and >23 were 0.707, 0.754, and 0.875. This result indicates that long-range contacts are more informative for distinguishing folds.

### Fold recognition with relaxed contact matching

We further examined fold recognition with a relaxed definition of common contacts. A pair of residue contacts in two proteins are considered as common when they occur within ±1 residues to each other in a given structural alignment. Although the results do not differ much from those by the original definition of common contacts, “blurring” contacts made fold recognition slightly worse for all three types of alignments. For TM-align alignments, AUC decreased from 0.907 to 0.888, from 0.847 to 0.837 for the HHpred alignments and from 0.717 to 0.699 for the SUPRB alignments. The AUC values are for the contact definition of Cβ-Cβ 6.5 Å.

### Fold recognition for different structural classes

**Figure 7:**
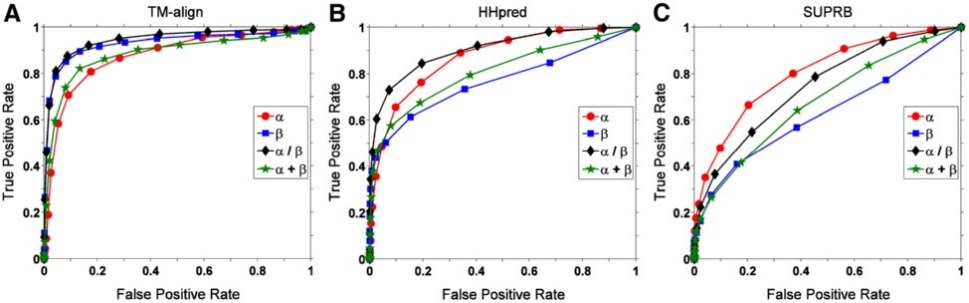
**ROC curves using TM-align, HHpred, and SUPRB for four different fold classes (α, β, α/β, and α+β).** The residue contact definition of the Cβ-Cβ distance 6.5Å was used. **A**, TM-align. AUC values for α, β, α/β and α+β are 0.876, 0.931, 0.945, and 0.866, respectively. **B**, HHpred. AUC values for the four classes are 0.871, 0.767, 0.904, and 0.808, respectively. **C**, SUPRB. AUC values are 0.802, 0.629, 0.745, and 0.679 for the four classes, respectively

**Figure 8:**
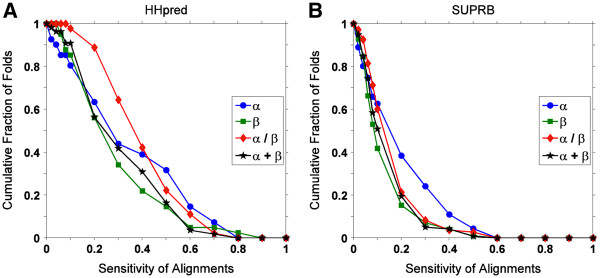
**Cumulative fraction of the alignment sensitivity for different fold class.** Alignments were computed using **A**, HHpred; and **B**, SUPRB. Protein pairs of the same fold were used. Alignment sensitivity was averaged over protein pairs of the same fold and the fraction of folds that have average alignment sensitivity above each cutoff was plotted

**Figure 9:**
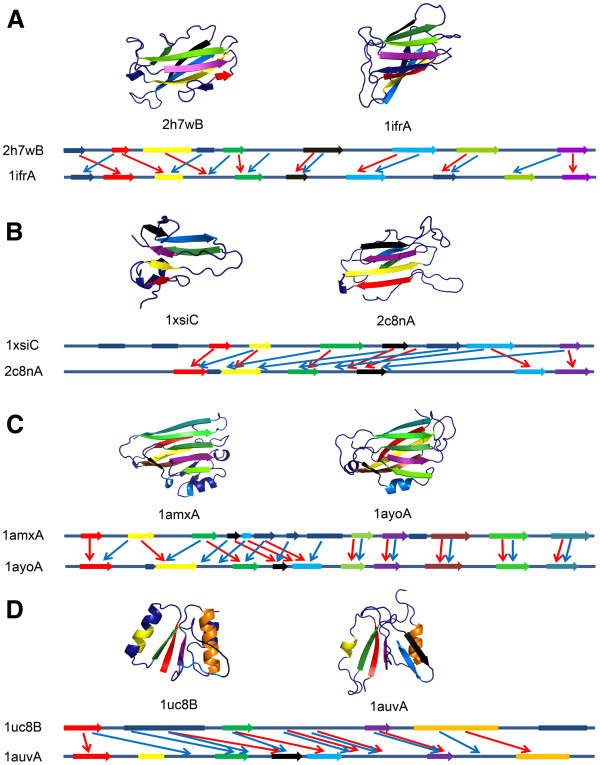
**Examples of structure pairs that are not correctly aligned by HHpred and/or SUPRB.** For each example, the structure pairs are colored to indicate corresponding secondary structures in a structural alignment by TM-align on the top panel. On the bottom panel, aligned secondary structures by HHpred and SUPRB are shown by red (HHpred) and blue (SUPRB) arrows. The secondary structures of the same color on the top and the bottom panel correspond to each other. Arrows indicate β strands while boxes are α helices. Note that the length of the lines does not reflect the actual size of the proteins (two proteins do not necessarily have the same length). **A**, left, 2h7wB (query), right 1ifrA (template). TM-score (RMSD) of the predicted structure of the query inferred from the HHpred/SUPRB alignments are 0.346 (4.20 Å) and 0.290 (4.09 Å), respectively. **B**, Left, 1xsiC (query), right, 2c8nA (template). TM-score (RMSD) of the structure models using HHpred/SUPRB alignments are 0.410 (2.83 Å) and 0.269 (3.45 Å), respectively. **C**, Left, 1amxA (query), right, 1ayoA (template). TM-score (RMSD) of the structure models using HHpred/SUPRB alignments are 0.591 (3.50 Å) and 0.589 (3.30 Å), respectively. **D**, Left, 1uc8_B (query), right, 1auvA (template). TM-score (RMSD) of the structure models using HHpred/SUPRB alignments are 0.438 (2.91 Å) and 0.416 (2.62 Å), respectively

Next, we examine the structure retrieval performance using FCC on four major fold classes separately, all-α class, all-β class, α/β class, and α + β class in the fold dataset (Figure 7). There are 41, 41, 45, and 55 folds in each class, respectively. We used the contact definition of 6.5Å for the Cβ-Cβ distance, since it is one of the best performing definitions in the previous experiments in Figures 1 and 5. Figure 7 shows that the structure retrieval performance varies for different fold class. When TM-align was used (Figure 7A), folds in the α/β, and the β class are better distinguished than those in the α+β and the α class. However, trend is different for retrieval using FCC computed with the two threading methods, HHpred (Figure 7B) and SUPRB (Figure 7C). Among the four classes, the all-β class performed worst. Since we found that the retrieval results for the threading methods are largely affected by their alignment accuracy (Figures 1, 2, 3), in Figure 8 we examined the alignment accuracy by HHpred (Figure 8A) and SUPRB (Figure 8B) for the four fold classes. It is found that, consistent to the retrieval results in Figure 7, all-β proteins show the lowest alignment accuracy by both HHpred and SUPRB. Moreover, the overall order of the accuracies for the four fold classes is consistent with the structure retrieval performance shown in Figure 7. Thus, it is confirmed that the retrieval accuracy of fold classes reflects the alignment accuracy of proteins in each classes. Figure 9 shows four examples of poorly recognized folds using FCC computed with the threading methods. The Cβ-Cβ distance 6.5 Å was used for the contact threshold. The first three pairs (Figures 9A, B, C) are from the all-β class while the last one is from the α/β class. Proteins in the first example (Figure 9A) have the immunoglobulin-like β-sandwich fold, which have two layers of β-sheets. The query (2h7wB) contains eight β strands and the template (1ifrA) has nine. TM-align aligned the two-layer structure correctly yielding an alignment with an RMSD of 4.21 Å for 72 residues, which corresponds to 67.3% and 63.7% of the length of 2h7wB and 1ifrA, respectively. The RMSD was computed by the LGA program [46]. On the other hand, the two threading methods shifted alignment at the N-terminal region (Figure 9A illustrates misalignments of HHpred and SUPRB), which resulted in RMSDs of 13.54 Å and 15.96 Å by HHpred and SUPRB, respectively. The FCC values by TM-align, HHpred, and SUPRB are 45. 8%, 2.2%, and 0.0%, respectively. The second example (Figure 9B) is proteins of the glycosyl hydrolase-fold. TM-align produced an alignment with a 3.15 Å RMSD and FCC of 50.0%, while the RMSDs of HHpred and SUPRB alignments are 12.16 Å and 12.29 Å with FCC of 38.8% and 13.8%, respectively. The third example (Figure 9C) is a protein pair of the “Common fold of diphtheria toxin/transcription factors/cytochrome f” fold, which have nine β strands forming two layers. TM-align captured the overall folds correctly with an RMSD of 3.10 Å and a FCC of 74.1%. On the other hand, HHpred and SUPRB misaligned the protein pair mainly at the first half of the proteins. This alignment error caused worse RMSDs of 5.79 Å and 16.74 Å and FCC values of 44.2%, and 9.9%, respectively. The last example is a protein pair in the α/β class (Figure 9D), the preATP-grasp domain fold. The structures of the proteins have a similar α/β/α three layer core. TM-align aligned each layer from the two proteins yielding an RMSD of 2.81 Å and FCC of 62.5%. However, HHpred and SUPRB shifted the whole alignment, resulting in RMSDs of 11.89 Å and 13.24 Å, and FCCs of 34.3% and 2.7%, respectively. These examples illustrate that the threading methods’ frequent mistakes of shifting β strands in their alignments, leading to failure of detecting conserved contact pattern of the proteins. 

### Best contact definitions for individual folds

**Figure 10:**
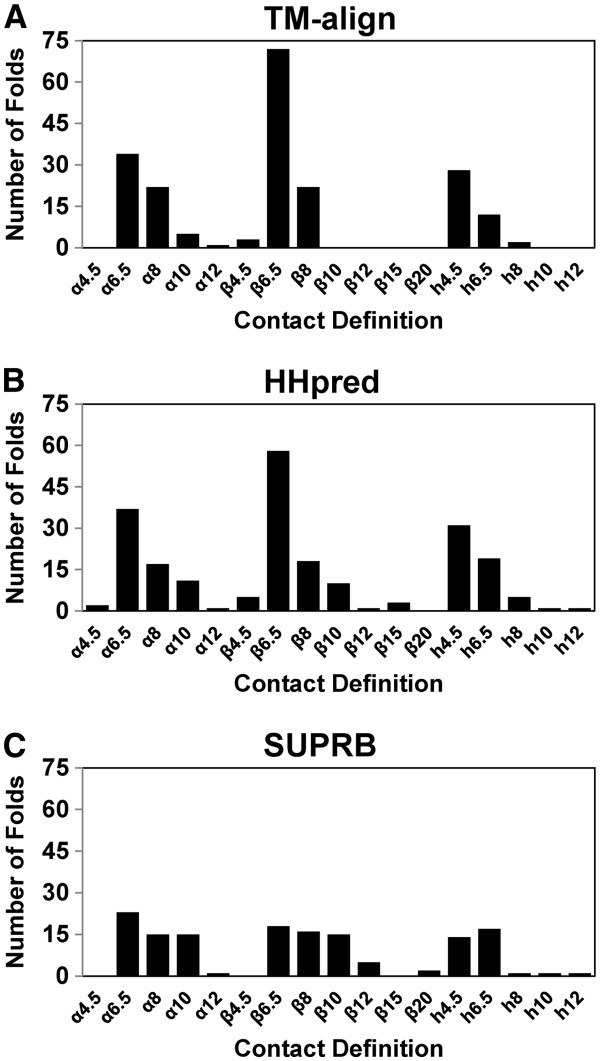
**Distribution of contact definitions for individual folds that give the largest AUC values.A**, TM-align, **B**, HHpred, and **C**, SUPRB. Folds are discarded if even the best contact definition has an AUC value less than 0.7. Among the thirty contact definitions examined, thirteen definitions are discarded which had zero counts for all the three alignment methods

**Figure 11:**
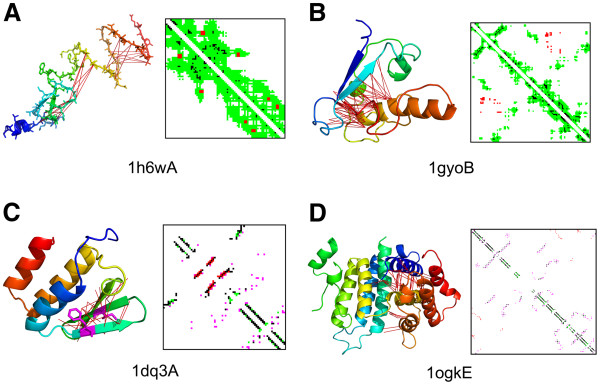
**Examples of folds that are better recognized by a contact definition different from the Cβ-Cβ distance 6.5Å.** For each example, the tertiary structure and a contact map are presented. Contact maps are generated using CMView [47]. The chain color, blue to red, shows the orientation of the chain from the N- to the C-terminus. **A**, 1h6wA. In the contact maps, black are contacts detected by both the Cβ-Cβ 6.5 Å and the Cβ-Cβ 20 Å definitions; green and red are contacts dentified only by the latter definition. Contacts in red in the map correspond to the residue pairs connected by red lines in the structure. **B**, 1gyoA. Black are contacts detected by both the Cβ-Cβ 6.5 Å and the Cα-Cα 12 Å; green and red are contacts identified only by the latter definition. **C**, 1dq3A. Black are identified by both the Cβ-Cβ 6.5Å and the Cα-Cα 6.5Å; red and green contacts are identified only by the latter definition. Two residues in magenta in the structure are in contact using the definition of the Cα-Cα 6.5 Å but not by the Cβ-Cβ 6.5 Å. **D**, 1ogkE. Black are those which identified by both the Cβ-Cβ 6.5 Å and the Cα-Cα 6.5 Å. On the other hand, contacts in purple and red are detected only by the former definition. Contacts in green are unique to the latter definition

Up to this point, we examined the fold retrieval performance of the various contact definitions averaged over all folds in the dataset. In this section, we investigate best contact definitions for individual protein folds. Different folds hold different conserved contact patterns. Thus, residue contact definitions that achieve the best fold recognition for a certain fold may be different from the 7.0 Å Cβ-Cβ distance that works best on average. For each fold, we selected a definition with the largest AUC value in the fold retrieval among 30 different definitions. If more than one definition has the same AUC value, then all of them are counted. A fold was not counted if its largest AUC is less than 0.7. Figure 10 shows the distributions of best performing contact definitions for folds for TM-align, HHpred, and SUPRB. When structure alignment by TM-align was used (Figure 10A), 6.5 Å Cβ-Cβ distance worked best in 72 out of 201 cases (35.8%). 6.5 Å Cα-Cα distance came to the second (best for 34 folds) and 4.5 Å heavy atom distance was the third (best for 28 folds). When HHpred was used for alignments (Figure 10B), larger distances that did not appear for TM-align (Figure 10A), i.e. 10 Å, 12 Å, and 15 Å for the Cβ-Cβ distance, performed best for some folds. For SUPRB (Figure 10C), the distribution of the contact definitions is very different from that of TM-align. Counts are more evenly distributed for different distance cutoffs. The largest counts were observed for the Cα-Cα distance 6.5 Å (23 cases; 16.0%), and the second and the third were the Cβ-Cβ distance 6.5 Å (18 cases) and the heavy atom distance 6.5 Å (17 cases). Why does the overall best definition not work well for some folds? Figure 11 illustrates when structures are better recognized by a contact definition that is different from the Cβ-Cβ distance 6.5Å. The first example, 1h6wA, has a loosely packed C-terminal region (Figure 11A). The best contact definition for this fold is the Cβ-Cβ distance of 20 Å when using TM-align. Such a large cutoff produces a contact map that contains all contacts from the definition of the Cβ-Cβ distance 6.5 Å and additionally captures neighboring residues in the C-terminus, as shown in the contact map. Although these “contacts” identified by the large distance cutoff are not physical interactions, characteristic structural information can be captured, which contributes for more accurate recognition of this fold. For the second example, 1gyoB, the best contact definition was found to be 12 Å for the Cα-Cα distance. As shown is Figure 11B, this definition covers all the contacts identified with the definition of Cβ-Cβ distance 6.5 Å as well as important interaction between α helices and β strands (e.g. contacts in red). The latter two examples show the difference of fold recognition abilities by the Cα-Cα distance 6.5 Å and the Cβ-Cβ distance 6.5 Å. The Cα-Cα distance of 6.5 Å had the second largest count for TM-align (Figure 10A) and HHpred (Figure 10B) and the largest count for SUPRB (Figure 10C). Figure 11C shows a structure of intein-encoded homing endonuclease PI-PfuI, ldq3A. The AUC value using the Cα-Cα distance 6.5 Å for this fold was 0.848 while it was significantly worse, 0.601, with the Cβ-Cβ distance of 6.5 Å when TM-align was used for the structure alignment. Contacts shown in red in the map are for residue pairs between three β strands (shown by red lines in the structure), which are detected only by using the Cα-Cα distance 6.5 Å. As illustrated by contacting residue pairs shown in magenta in the structure figure, these contacts are not detected by the Cβ-Cβ distance 6.5 Å because the side-chains are placed in opposite directions. In contrast, the Cβ-Cβ distance 6.5 Å performed better for capturing residue contacts between α helices for 1ogkE (Figure 11D). AUC using the Cβ-Cβ distance 6.5 Å and the Cα-Cα distance 6.5 Å for structural alignments were 0.97 and 0.92, respectively. Contacts colored in red in both the structure and the contact map are those which are captured uniquely by the Cβ-Cβ distance 6.5 Å but not Cα-Cα distance 6.5 Å because they are too far for the latter.

## Discussion

In this work, we tested thirty different residue contact definitions in the context of fold recognition. To investigate the pure ability of contact patterns for distinguishing folds, we introduced the fraction of common contacts (FCC) of protein pairs and examined how well FCC computed with different definitions select proteins of the same fold from the rest of the protein pairs of different folds. To examine how much incorrect alignments in threading affect the fold recognition accuracy, we also used two threading methods, HHpred and SUPRB, to determine corresponding residues of proteins. We found that overall, the Cβ-Cβ distance 7.0 Å works best for identifying proteins of the same fold consistently for structural alignments and threading alignments. A qualitative difference between the threading alignments and structural alignments is that the former prefer larger distance cutoffs for defining contacts because they are more tolerant to misalignments (Figure 10). In the case of threading, alignment accuracy strongly influences the fraction of common contacts identified among proteins of the same fold (Figure 2), which eventually affects fold recognition accuracy (Figures 1, 5). It turned out that threading alignment accuracy is relatively poorer for all-β proteins (Figure 8), and thus those proteins have lower fold recognition accuracy (Figure 7). Finally, we found that the effective contact definition to identify folds depends on the folds (Figure 10). A larger distance cutoff is advantageous for capturing spatial arrangement of the secondary structures of a fold, which are not physically in contact. For capturing contacts between neighboring β strands, considering Cα atoms is better than Cβ, because sometimes the side-chains point to opposite directions (Figure 11C). The results of this work suggest two potential directions of implementing residue contacts for improving fold recognition. Since a larger distance cutoff is effective in capturing local topology of proteins, employing a “long-distance” interaction potential for residues that are 6.5 Å to 12 Å apart may improve recognition accuracy. The long-distance interaction potential may be used as a scoring term in threading together with a regular contact potential (e.g. for contacts defined within 4.5 Å between heavy atoms). Another idea is to use different fold-specific contact definitions (Figures 10, 11) for each structure in a template database.

## Conclusions

This study focused on seeking effective inter-residue contact definitions for template-based protein structure prediction. Residue contacts defined by Cβ−Cβ distance of 7.0 Å work best overall among tested to identify proteins of the same fold. We also found that effective contact definitions differ from fold to fold, suggesting that using different residue contact definition specific for each template will lead to improvement of the performance of threading.

## Methods

### Dataset of domain structures of globular proteins

Two sets of domain structures of globular proteins were selected according to the SCOP database (release 1.73) [48], one for representative protein folds and another one for representative superfamilies. We selected protein folds that have at least three superfamilies, from each of which one domain structure was selected. Entries were discarded if their PDB files contain only Cα traces. In total, 194 folds were selected. The numbers of structures in each fold range from 3 to 110. In total, there are 2167 structures in the fold dataset. Similarly, a dataset of 250 representative superfamilies that contains a total of 1672 structures were selected. Each superfamily in the dataset contains at least three families, from each of which one structure was selected. In the following part, we will explain the experiment procedure on the fold dataset and readers should be aware that the same procedure was performed on the superfamily dataset.

### Construction of contact maps

For each structure in the datasets, we constructed contact maps using thirty different contact definitions: three contact bases to consider for an amino acid residue, i.e. Cα, Cβ (Cα atom is used for glycine), and heavy atoms from two residues, with 15 distance cutoffs for each (4.5, 5.0, 5.5, 6.0, 6.5, 7.0, 7.5, 8.0, 10.0, 12.0, 15.0, 20.0, 30.0, 50.0, and 100.0 Å). To eliminate obvious contacts from neighboring residues, we only considered contacts between amino acid residues that are at least three residues apart in the primary sequence.

### Common contacts between two protein structures

The aim of this work is to examine how well residue contacts determined by each of thirty definitions can distinguish proteins of the same fold from the others. To identify common contacts between two protein structures (more precisely, contact maps of the two protein structures), we need an alignment of the two proteins to identify structurally equivalent residues between them. Alignments were obtained using three methods, TM-align [43], HHpred [44], and SUPRB [12]. TM-align is a structure alignment method, which aligns two tertiary structures using a dynamic programming algorithm and computes the root mean square deviation (RMSD). We consider structural alignments calculated by TM-align as the golden standard of the alignments. The latter two methods, HHpred and SUPRB, are threading methods. For a pair of proteins, the sequence of one of them is threaded (aligned) on the other protein structure. The purpose of using the threading methods is to introduce realistic errors that can happen in the alignment process of threading. HHpred uses a hidden Markov model that characterizes proteins with sequence profiles and predicted secondary structures [44]. SUPRB is a threading method that uses a composite scoring function with sequence profile, solvent accessibility, secondary structure matching, main chain angle preference, and a residue contact potential term. In this experiment we deleted the contact potential term from the scoring function. Given contact maps of two proteins and an alignment (either by TM-align, HHPred, or SUPRB), the fraction of common contacts (FCC) was computed as follows: Suppose residues *a*_*i*_ and *a*_*j*_ in protein A (the query) are aligned with residues *b*_*m*_ and *b*_*n*_ in another protein B (the template), respectively. If the (*a*_*i*_ , *a*_*j*_) pair and the *(b*_*m*_*, b*_*n*_*)* pair are in contact within each protein respectively, then we count them as a common contact between the two proteins. Finally, the FCC for the query protein is computed as the number of residues in the query that are involved in at least one common contact relative to the number of aligned residues. FCC ranges from 0 to 1.

### Identification of proteins of the same fold/superfamily by fraction of common contacts

For a group of proteins of the same fold, FCC was computed for each pair of them. As a reference, we took one protein from each fold (thus 194 proteins in total) and computed FCC between the selected protein of the fold and the other proteins from different folds. The difference between FCC values of proteins within the same fold and those across different folds reflects the ability of fold recognition by a certain definition of residue contacts. For a fold group, we sorted protein pairs of the same fold and those from different folds by their FCC and computed the receiver operator characteristic (ROC) curve. For each contact definition, an average ROC curve was computed by averaging the true positive values of all the folds at the same false positive rate.

## Competing interests

The authors declare that they have no competing interests.

## Authors’ contributions

CY participated in design, helped implementation of programs for the study, performed the analysis, and drafted the paper. HC coded some of the programs used and prepared the dataset. DK conceived of the study, participated in its design, and finalized the manuscript. All authors read and approved the final manuscript.

## Supplementary Material

Additional file 1 **ROC curves of structure pair retrieval on the superfamily dataset.** To determine corresponding residues in protein pairs, TM-align (the left column), HHpred (the middle column), and SUPRB (the right column) were used. Three different bases for residue contact definitions are used, the Cα-Cα (the first row), the Cβ-Cβ distance (the second row), and the heavy atom distance (the third row). The panels are ordered in the same way as in Figure 1. Click here for file

## References

[B1] Hillisch A Pineda LF Hilgenfeld R Utility of homology models in the drug discovery process Drug Discov Today 2004 9 659 669 10.1016/S1359-6446(04)03196-4 15279849 PMC7129151

[B2] Takeda-Shitaka M Takaya D Chiba C Tanaka H Umeyama H Protein structure prediction in structure based drug design Curr Med Chem 2004 11 551 558 10.2174/0929867043455837 15032603

[B3] Ashworth J Havranek JJ Duarte CM Sussman D Monnat RJ Jr Stoddard BL Baker D Computational redesign of endonuclease DNA binding and cleavage specificity Nature 2006 441 656 659 10.1038/nature04818 16738662 PMC2999987

[B4] Jiang L Althoff EA Clemente FR Doyle L Rothlisberger D Zanghellini A Gallaher JL Betker JL Tanaka F Barbas CF III Hilvert D Houk KN Stoddard BL Baker D De novo computational design of retro-aldol enzymes Science 2008 319 1387 1391 10.1126/science.1152692 18323453 PMC3431203

[B5] Saven JG Computational protein design: engineering molecular diversity, nonnatural enzymes, nonbiological cofactor complexes, and membrane proteins Curr Opin Chem Biol 2011 15 452 457 10.1016/j.cbpa.2011.03.014 21493122 PMC4077665

[B6] Mardis ER Next-generation DNA sequencing methods Annu Rev Genomics Hum Genet 2008 9 387 402 10.1146/annurev.genom.9.081307.164359 18576944

[B7] Metzker ML Sequencing technologies - the next generation Nat Rev Genet 2010 11 31 46 10.1038/nrg2626 19997069

[B8] Berman HM Westbrook J Feng Z Gilliland G Bhat TN Weissig H Shindyalov IN Bourne PE The protein data bank Nucleic Acids Res 2000 28 235 242 10.1093/nar/28.1.235 10592235 PMC102472

[B9] Pieper U Eswar N Davis FP Braberg H Madhusudhan MS Rossi A Marti-Renom M Karchin R Webb BM Eramian D Shen MY Kelly L Melo F Sali A MODBASE: a database of annotated comparative protein structure models and associated resources Nucleic Acids Res 2006 34 D291 D295 10.1093/nar/gkj059 16381869 PMC1347422

[B10] Kihara D Skolnick J Microbial Genomes have over 72% structure assignment by the threading algorithm PROSPECTOR_Q Proteins 2004 55 464 473 10.1002/prot.20044 15048836

[B11] Zhang Y Progress and challenges in protein structure prediction Curr Opin Struct Biol 2008 18 342 348 10.1016/j.sbi.2008.02.004 18436442 PMC2680823

[B12] Chen H Kihara D Effect of using suboptimal alignments in template-based protein structure prediction Proteins 2011 79 315 334 10.1002/prot.22885 21058297 PMC3058269

[B13] Kinch L Yong SS Cong Q Cheng H Liao Y Grishin NV CASP9 assessment of free modeling target predictions Proteins 2011 79 Suppl 10 59 73 21997521 10.1002/prot.23181 PMC3226891

[B14] Qu X Swanson R Day R Tsai J A guide to template based structure prediction Curr Protein Pept Sci 2009 10 270 285 10.2174/138920309788452182 19519455

[B15] Liu S Zhang C Liang S Zhou Y Fold recognition by concurrent use of solvent accessibility and residue depth Proteins 2007 68 636 645 10.1002/prot.21459 17510969

[B16] Zhou H Zhou Y Single-body residue-level knowledge-based energy score combined with sequence-profile and secondary structure information for fold recognition Proteins 2004 55 1005 1013 10.1002/prot.20007 15146497

[B17] Skolnick J Kihara D Defrosting the frozen approximation: PROSPECTOR–a new approach to threading Proteins 2001 42 319 331 10.1002/1097-0134(20010215)42:3<319::AID-PROT30>3.0.CO;2-A 11151004

[B18] Skolnick J Kihara D Zhang Y Development and large scale benchmark testing of the PROSPECTOR 3.0 threading algorithm Proteins 2004 56 502 518 10.1002/prot.20106 15229883

[B19] Adamczak R Porollo A Meller J Combining prediction of secondary structure and solvent accessibility in proteins Proteins 2005 59 467 475 10.1002/prot.20441 15768403

[B20] Yang YD Park C Kihara D Protein structure prediction without optimizing weighting factors for scoring function Biophys J 2009 96 653a 10.1002/prot.22082 18473394

[B21] Sippl MJ Knowledge-based potentials for proteins Curr Opin Struct Biol 1995 5 229 235 10.1016/0959-440X(95)80081-6 7648326

[B22] Skolnick J Jaroszewski L Kolinski A Godzik A Derivation and testing of pair potentials for protein folding. When is the quasichemical approximation correct Protein Sci 1997 6 676 688 9070450 10.1002/pro.5560060317 PMC2143667

[B23] Zhou H Skolnick J GOAP: a generalized orientation-dependent, all-atom statistical potential for protein structure prediction Biophys J 2011 101 2043 2052 10.1016/j.bpj.2011.09.012 22004759 PMC3192975

[B24] Kihara D The effect of long-range interactions on the secondary structure formation of proteins Protein Sci 2005 14 1955 1963 10.1110/ps.051479505 15987894 PMC2279307

[B25] Taketomi H Ueda Y Go N Studies on protein folding, unfolding and fluctuations by computer simulation. I. The effect of specific amino acid sequence represented by specific inter-unit interactions Int J Pept Protein Res 1975 7 445 459 1201909

[B26] Vassura M Di LP Margara L Mirto M Aloisio G Fariselli P Casadio R Blurring contact maps of thousands of proteins: what we can learn by reconstructing 3D structure BioData Min 2011 4 1 10.1186/1756-0381-4-1 21232136 PMC3033854

[B27] Duarte JM Sathyapriya R Stehr H Filippis I Lappe M Optimal contact definition for reconstruction of contact maps BMC Bioinformatics 2010 11 283 10.1186/1471-2105-11-283 20507547 PMC3583236

[B28] Vendruscolo M Kussell E Domany E Recovery of protein structure from contact maps Fold Des 1997 2 295 306 10.1016/S1359-0278(97)00041-2 9377713

[B29] Li W Zhang Y Kihara D Huang YJ Zheng D Montelione GT Kolinski A Skolnick J TOUCHSTONEX: protein structure prediction with sparse NMR data Proteins 2003 53 290 306 10.1002/prot.10499 14517980

[B30] Rodionov MA Johnson MS Residue-residue contact substitution probabilities derived from aligned three-dimensional structures and the identification of common folds Protein Sci 1994 3 2366 2377 10.1002/pro.5560031221 7756991 PMC2142768

[B31] Li Y Fang Y Fang J Predicting residue-residue contacts using random forest models Bioinformatics 2011 27 3379 3384 10.1093/bioinformatics/btr579 22016406

[B32] Shackelford G Karplus K Contact prediction using mutual information and neural nets Proteins 2007 69 Suppl 8 159 164 17932918 10.1002/prot.21791

[B33] Frenkel-Morgenstern M Magid R Eyal E Pietrokovski S Refining intra-protein contact prediction by graph analysis BMC Bioinformatics 2007 8 Suppl 5 S6 10.1186/1471-2105-8-S5-S6 17570865 PMC1892094

[B34] Cheng J Baldi P Improved residue contact prediction using support vector machines and a large feature set BMC Bioinformatics 2007 8 113 10.1186/1471-2105-8-113 17407573 PMC1852326

[B35] Hamilton N Burrage K Ragan MA Huber T Protein contact prediction using patterns of correlation Proteins 2004 56 679 684 10.1002/prot.20160 15281121

[B36] Fariselli P Olmea O Valencia A Casadio R Prediction of contact maps with neural networks and correlated mutations Protein Eng 2001 14 835 843 10.1093/protein/14.11.835 11742102

[B37] Vullo A Walsh I Pollastri G A two-stage approach for improved prediction of residue contact maps BMC Bioinformatics 2006 7 180 10.1186/1471-2105-7-180 16573808 PMC1484494

[B38] Kihara D Lu H Kolinski A Skolnick J TOUCHSTONE: an ab initio protein structure prediction method that uses threading-based tertiary restraints Proc Natl Acad Sci U S A 2001 98 10125 10130 10.1073/pnas.181328398 11504922 PMC56926

[B39] Miyazawa S Jernigan RL An empirical energy potential with a reference state for protein fold and sequence recognition Proteins 1999 36 357 369 10.1002/(SICI)1097-0134(19990815)36:3<357::AID-PROT10>3.0.CO;2-U 10409829

[B40] Miyazawa S Jernigan RL Estimation of effective inter-residue contact energies from protein crystal structures: quasi-chemical approximation Macromolecules 1985 18 534 552 10.1021/ma00145a039

[B41] Gniewek P Leelananda SP Kolinski A Jernigan RL Kloczkowski A Multibody coarse-grained potentials for native structure recognition and quality assessment of protein models Proteins 2011 79 1923 1929 10.1002/prot.23015 21560165 PMC3093657

[B42] Krishnamoorthy B Tropsha A Development of a four-body statistical pseudo-potential to discriminate native from non-native protein conformations Bioinformatics 2003 19 1540 1548 10.1093/bioinformatics/btg186 12912835

[B43] Zhang Y Skolnick J TM-align: a protein structure alignment algorithm based on the TM-score Nucleic Acids Res 2005 33 2302 2309 10.1093/nar/gki524 15849316 PMC1084323

[B44] Hildebrand A Remmert M Biegert A Soding J Fast and accurate automatic structure prediction with HHpred Proteins 2009 77 Suppl 9 128 132 19626712 10.1002/prot.22499

[B45] Xu J Zhang Y How significant is a protein structure similarity with TM-score = 0.5 Bioinformatics 2010 26 889 895 10.1093/bioinformatics/btq066 20164152 PMC2913670

[B46] Zemla A LGA: A method for finding 3D similarities in protein structures Nucleic Acids Res 2003 31 3370 3374 10.1093/nar/gkg571 12824330 PMC168977

[B47] Vehlow C Stehr H Winkelmann M Duarte JM Petzold L Dinse J Lappe M CMView: interactive contact map visualization and analysis Bioinformatics 2011 27 1573 1574 10.1093/bioinformatics/btr163 21471016

[B48] Andreeva A Howorth D Chandonia JM Brenner SE Hubbard TJ Chothia C Murzin AG Data growth and its impact on the SCOP database: new developments Nucleic Acids Res 2008 36 D419 D425 18000004 10.1093/nar/gkm993 PMC2238974

