# Supplementary material for: Effective inter-residue contact definitions for accurate protein fold recognition
Source: BMC Bioinformatics. 2012 Nov 9;13:292. doi: 10.1186/1471-2105-13-292 (PMC3534397; doi:10.1186/1471-2105-13-292)
Supplement: Additional file 1 — ROC curves of structure pair retrieval on the superfamily dataset. To determine corresponding residues in protein pairs, TM-align (the left column), HHpred (the middle column), and SUPRB (the right column) were used. Three different bases for residue contact definitions are used, the Cα-Cα (the first row), the Cβ-Cβ distance (the second row), and the heavy atom distance (the third row). The panels are ordered in the same way as in Figure 1. [file 1471-2105-13-292-S1.pdf]

Supplemental Material for

Effective Inter-Residue Contact Definitions for Accurate Protein Fold Recognition

Chao Yuan, Hao Chen, and Daisuke Kihara

Contact: dkihara@purdue.edu

Figure S1

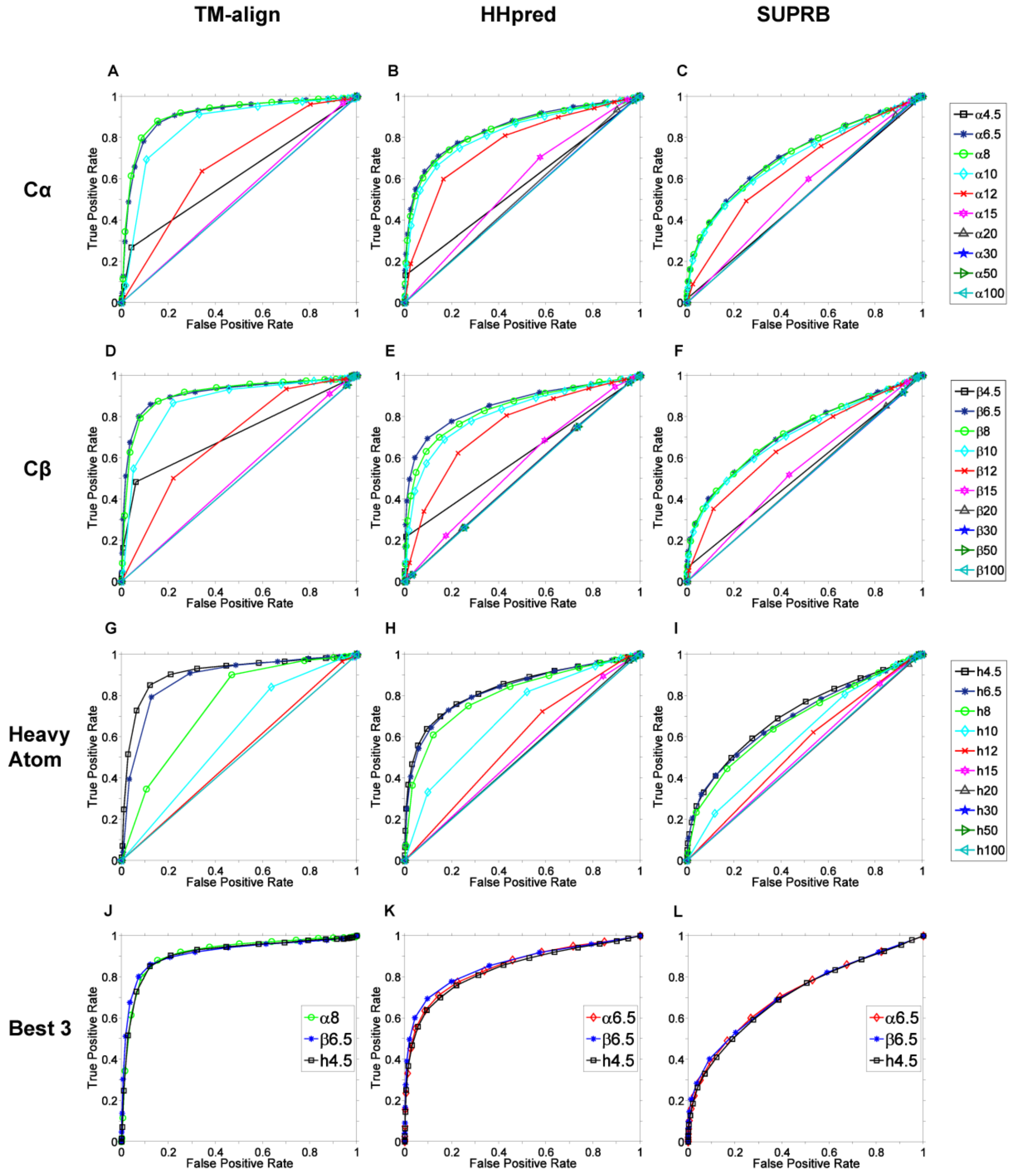

Figure 1. ROC curves of structure pair retrieval on the superfamily dataset. To determine corresponding residues in protein pairs, TM-align (the left column), HHpred (the middle column), and SUPRB (the right column) were used. Three different bases for residue contact definitions are used, the Cα-Cα (the first row), the Cβ-Cβ distance (the second row), and the heavy atom distance (the third row). The panels are ordered in the same way as in Figure 1.
